# Mapping the Evolution of Activity-Based Protein Profiling: A Bibliometric Review

**DOI:** 10.34172/apb.2023.082

**Published:** 2023-05-20

**Authors:** Exequiel Oscar Jesus Porta

**Affiliations:** Department of Chemistry, Durham University, Durham, DH1 3LE, United Kingdom.

**Keywords:** Activity-based protein profiling, Activity-based probes, Affinity-based probes, Bibliometric analysis, Chemical probes, Drug discovery

## Abstract

Activity-based protein profiling (ABPP) is a chemoproteomic approach that employs small-molecule probes to directly evaluate protein functionality within complex proteomes. This technology has proven to be a potent strategy for mapping ligandable sites in organisms and has significantly impacted drug discovery processes by enabling the development of highly selective small-molecule inhibitors and the identification of new therapeutic molecular targets. Despite being nearly a quarter of a century old as a chemoproteomic tool, ABPP has yet to undergo a bibliometric analysis. In order to gauge its scholarly impact and evolution, a bibliometric analysis was performed, comparing all 1919 reported articles with the articles published in the last five years. Through a comprehensive data analysis, including a 5-step workflow, the most influential articles were identified, and their bibliometric parameters were determined. The 1919 analyzed articles span from 1999 to 2022, providing a comprehensive overview of the historical and current state of ABPP research. This analysis presents, for the first time, the characteristics of the most influential ABPP articles, offering valuable insight into the research conducted in this field and its potential future directions. The findings underscore the crucial role of ABPP in drug discovery and novel therapeutic target identification, as well as the need for continued advancements in the development of novel chemical probes and proteomic technologies to further expand the utility of ABPP.

## Introduction

 Activity-based protein profiling (ABPP) is a chemoproteomic technology that has revolutionized the field of drug discovery by enabling the identification of proteins that can be targeted by small molecules. ABPP achieves this by utilizing activity-based probes (ABPs) that covalently label active proteins within complex proteomes. By combining ABPs with proteomics tools, ABPP provides a powerful means of studying protein functionality and mapping ligandable sites. This approach has significantly impacted drug discovery processes by enabling the development of highly selective small-molecule inhibitors and the identification of new therapeutic targets. One of the key advantages of ABPP is that it allows us to study the mode of action of compounds in their native environment, overcoming one of the major challenges in the field of drug discovery.^1^ ABPs are designed to selectively label the active forms of enzymes within complex proteomes, enabling the characterization of changes in enzyme activity that occur without alterations in protein levels. This feature allows for a more accurate assessment of enzyme function and provides valuable insights into the regulation of biological pathways. This advantage positions ABPP as an interesting tool, compared to conventional gene expression and other proteomic methods, to discover new enzymes.^2^

**Figure 1:**
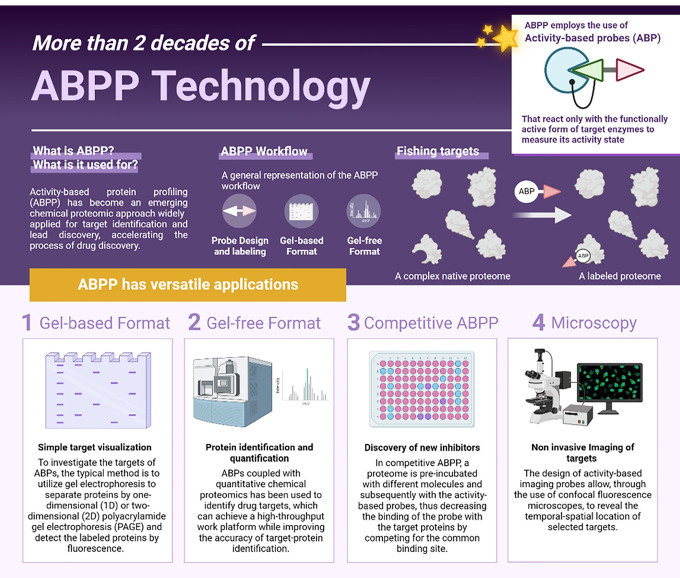
Main concepts and applications of ABPP. Since its development, ABPP has become a versatile tool for the discovery of new targets and drugs

 ABPP-like experiments were initially conducted in the 1970s to study the mechanisms of penicillin,^3^ but it was not until the late 1990s that the modern era of ABPP began with the development of ABPs that were compatible with proteomics techniques. The term “ABPP” was coined for the first time in 1999,^4^ and since then, the number of publications on ABPP has grown exponentially, reflecting the rapid evolution of this powerful chemoproteomic technology. ABPP has been successfully applied to study a wide range of biological processes and diseases, making it a valuable tool for both basic research and drug discovery. Recent advances in proteomics have expanded the scope of ABPP, enabling its application in a variety of areas, from the study of different enzyme classes, such as kinases,^5^ proteases,^6^ glycosidases,^7^ phosphatases,^8^ and oxidoreductases,^9^ to cell imaging^10^ (Figure 1). ABPP has significantly enhanced our understanding of enzyme activity in various physiological and pathological processes on a proteome-wide scale.^11^

 On the other hand, bibliometric analyses have emerged as one of the most extensively used methods to measure the impact, quality, and credibility of scholarly articles.^12^ One of the most applied approaches for this analysis includes citation frequency, which reports the number of times that the article has been cited.^13^ Therefore, the most frequently cited articles are, arguably, the ones making the highest impact on its scientific community. Although not an infallible technique, bibliometric analysis can serve as a useful tool to identify potential (underexplored) areas of research within a discipline and to guide the allocation of resources by funding agencies.^14^

 By providing a comprehensive overview of contributors, hot topics, keywords, and citation performance, bibliometric analyses have become an essential tool for assessing the global impact of a particular research field. Despite its significance as a chemoproteomic technology, no such analysis of ABPP has been previously conducted. Therefore, the present study aims to bridge this gap by employing a 5-step roadmap to identify and evaluate ABPP-related publications in the existing scientific literature. By providing a quantitative assessment of the scholarly impact and evolution of ABPP, this analysis aims to offer valuable insights into the current state of the field and its potential future directions.

## Step 1: The primary definition of the search criteria, as well as the database used, is critical to start the data mining.

 On January 1st, 2023, a comprehensive literature search was conducted using the Web of Science (WoS) online database (Clarivate Analytics, Philadelphia, PA, USA). The search strategy employed included the following keywords: TOPIC = [“Activity base* prot*” (All Fields) or “Affinity base* prot*” (All Fields) or “Activity base* prob*” (All Fields) or “Affinity base* prob*” (All Fields)]. WoS was used as the main database because of its broader coverage of journals compared with other databases.^15^ No limitations were placed on publication year, language, or type of publication (e.g., original research article, review, editorial, book chapter, etc.) during the literature search.

## Step 2: Data mining requires a careful manual curation to minimize bias. Then, the generated database will allow the adequate data analysis.

 Articles were first assessed for their suitability for inclusion by title, abstract, and keywords. In case of any ambiguity, full texts were also reviewed. The generated databases (all articles and last 5-year articles) were re-evaluated and recorded taking into consideration: type of publication, publication year, journal title, total citation count, authorship, etc. The databases were imported into MS Excel and VOSviewer 1.6.18 (CWTS, Leiden University, Leiden, The Netherlands)^16^ for bibliometric analyses, such as citations, performances of institutions, countries/regions, journals, among others.

## Step 3: Direct bibliometric analysis reveals different metrics to be examined and compared, providing a profile on ABPP research.

**Figure 2:**
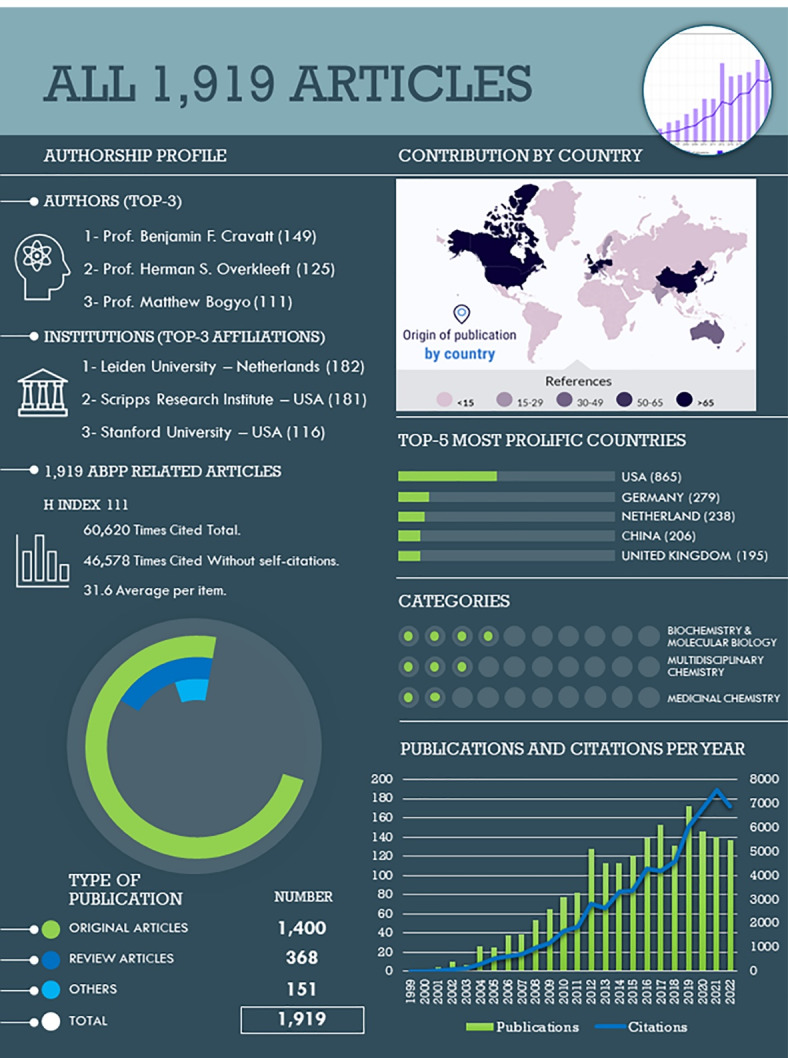
Bibliographic analysis of all ABPP-related articles. In detail: top-3 author contributions, top-3 institutions, top-5 countries, top-3 categories, all types of publications, total citations (1999-2022), and publications and citations per year

 A total of 1,919 publications were identified and analyzed(Figure 2). The first article, where the technique is named for first time as “ABPP”, was published in 1999 by Liu et al,^4^ laying the groundwork for further studies. It is, in fact, the fifth most-cited publication in this field (792 citations). Since then, ABPP was widely accepted by the scientific community, as evidenced by its upward trend in the number of articles reported, as well as in the number of total citations. As an illustration of that, it has taken 4 years to break the 100 citations per year mark, but only 9 years to break the 1000 citations per year mark. The number of reported publications has grown steadily, with a peak of 172 publications in 2019. The SARS-CoV-2 pandemic has stopped this increasing pace, but this phenomenon has also been observed in other scientific areas.^17^

 Within the total of 1919 publications in the field of active-based probes and affinity-based probes (AfBPs), there were 1400 original articles (73.0%) and 368 review articles (19.2%). The remaining 7.9% of the publications included meeting abstracts, proceeding papers, and editorial materials. Together, these articles were cited 60 620 times (46 578 times discounting self-citations that represented 23.2% of total), with an average of 31.6 citations per article and an H Index of 111. Within the WoS science categories, the publications were mainly classified as biochemistry and molecular biology (733; 38.2%), multidisciplinary chemistry (457; 23.8%), and medicinal chemistry (289; 15.1%), as the three most prolific categories. Three authors have more than 100 publications in this field, that is, Prof. Benjamin F. Cravatt (149; 7.8%; Scripps Research Institute, USA), Prof. Herman S. Overkleeft (125; 6.5%; Universiteit Leiden, the Netherlands), and Prof. Matthew Bogyo (111; 5.8%; Stanford University, USA). The four most prolific institutions have each accounted for 5% of total publications, namely, Leiden University (182; 9.5%; Netherlands), the Scripps Research Institute (181; 9.4%; USA), Stanford University (116; 6.0%; USA), and the University of California (97; 5.1%; USA). Consistently, USA (865; 45.1%), Germany (279; 14.5%), Netherland (238; 12.4%), People’s Republic of China (206; 10.7%), and the United Kingdom (195; 10.2%) were the leading countries in publishing ABPP-related articles. Meanwhile, the top 5 prolific journals were the Journal of the American Chemical Society (86; 4.5%; Impact Factor or IF = 16.38), ChemBioChem (76; 4.0%; IF = 3.46), ACS Chemical Biology (76; 4.0%; IF = 4.63), Angewandte Chemie International Edition (75; 3.9%; IF = 16.82), and Chemical Communication (47; 2.5%; IF = 6.06).

 In order to visualize the progress of this methodology, it is necessary to understand the changes that have been made during the last five years. To achieve this, an extra sub-database containing the articles published in the last 5 years (2018-2022; 726 publications) was extracted from the all-article database. This subset represented the 37.8% of total publications. Out of the 726 publications, 544 were original articles (74.9%) and 152 were review articles (20.9%). These articles received a total of 5,765 citations (5,374 when excluding self-citations, which accounted for 6.8% of the total), with an average of 10.8 citations per article and an H-Index of 38. The top three WoS science categories were biochemistry and molecular biology (258; 35.5%), multidisciplinary chemistry (190; 26.2%), and medicinal chemistry (115; 15.8%). Five authors stand out, having published over 20 articles in this field: Prof. Herman S. Overkleeft (40; 5.5%; Universiteit Leiden, the Netherlands), Prof. Johannes M. Aerts (22; 3.0%; Universiteit Leiden, the Netherlands), Prof. Benjamin F. Cravatt (21; 2.9%; Scripps Research Institute, USA), Prof. Matthew Bogyo (21; 2.9%; Stanford University, USA), and Prof Marcin Drag (21, 2.9%, University of Wrocław, Poland). Only one institution accounts for more than 5% of total publications, namely Leiden University (85; 11.7%; Netherlands). The leading countries in publishing ABPP-related articles were the USA (285; 39.3%), the People’s Republic of China (136; 18.7%), the Netherlands (107; 14.7%), Germany (104; 14.3%), and the United Kingdom (100; 13.8%). The five most prolific journals in this field were the ACS Chemical Biology (39; 5.3%; IF = 4.63), Journal of the American Chemical Society (28; 3.9%; Impact Factor = 16.38), Angewandte Chemie International Edition (28; 3.9%; IF = 16.82), ChemBioChem (27; 3.7%; IF = 3.46), and Journal of Medicinal Chemistry (21; 2.9%; IF = 8.04).

## Step 4: Mapping and correlating data allows us to visualize the big picture of this field.

**Figure 3:**
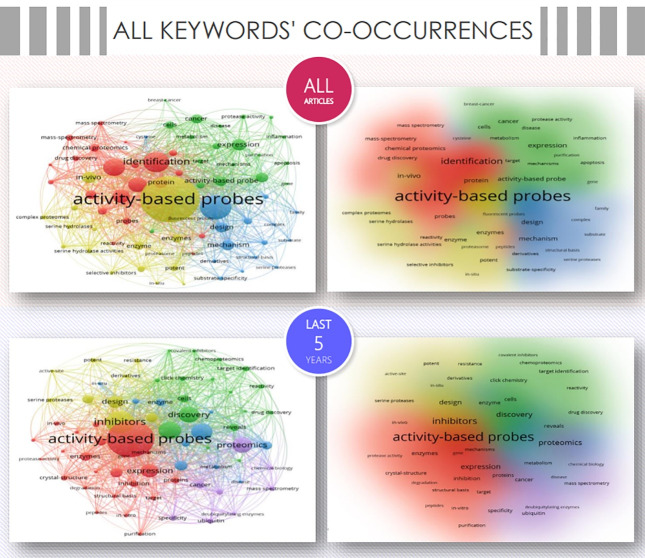
All-keyword co-occurrence networks and densities. Keywords, which appeared more than 30 times for all-articles and more than 12 times for the last-5-years databases, were presented in the co-occurrence networks. Within the networks, each circle represented a keyword, and the size of its circle represented the occurrence time. Circles in the same color are keywords included in the same cluster. The circles are connected only if the keywords appear in the same article, while the thickness of the line represents the number of publications in which they appear together. During the analysis, words with the same meaning were merged, and worthless words were manually excluded. A total of 4 clusters were found for the analysis of all articles, namely: new target(s) and drug discovery by mass spectrometry (red), diseases (green), serine hydrolases (yellow), and substrate-enzyme mechanisms (blue). For the subset of the last 5-year ABPP-related articles, five cluster were noted: resistance mechanisms (yellow), target identification and drug discovery (green), metabolism studies (blue), ubiquitylation system (violet), and enzymatic mechanisms (red)

 The VOSviewer was applied to map the co-occurrence network of all keywords, that is, author keywords and keywords plus (Figure 3). Given that keywords are a tool to help indexers and search engines find relevant articles, they constitute a key piece to analyze the current and future perspectives in the field.^18^

 A total of 7124 all keywords were reported for all articles, of which “activity-based probe(s)” (675), “identification/discovery” (415), “inhibitor(s)” (363), “activity-based protein profiling” (275), “proteomics” (252), stand out as the top 5 most-used. The network was organized in 4 clusters, when all 77 keywords with more than 30 co-occurrences were analyzed.

 When considering the last 5-year articles (3,537 all keywords), the top 5 most-representative keywords were “activity-based probe(s)” (207), “inhibitor(s)” (160), “identification/discovery” (151), “activity-based protein profiling” (84), and “proteomics” (74). In particular, when analyzing the 67 keywords ( > 12 co-occurrences) were grouped into 5 clusters, where the research areas oriented to the ubiquitinoylation system,^19^ and the understanding of resistance mechanisms^20^ stand out and appear as the areas of greatest growth in ABPP.

## Step 5: Comprehensive analysis of all generated data produce an overview on the current and future directions in ABPP research.

 ABPP has become an emerging chemoproteomic approach that has grown steadily during all these years, reaching its maximum of publications in 2019; however, this mark could be broken in the coming years.^21^ An interesting aspect to highlight is the universalization of the technique that can be evidenced in the relative weight of the USA in the publications during the last 5 years (285 documents; 39.5%), compared to 45.1% of the total of articles, and the rise of, for example, the People’s Republic of China from 10.7% for all publications to 18.7% in the last 5 years.

 The wide application of ABPP has been firmly established in several areas,^22^ as can be seen in the diversity of WoS categories. In the future, the combination of ABPP (and new ABPs) and novel chemical proteomic technologies will be the most important focus, expanding the universe of the druggable proteome in several organisms and providing more opportunities to accelerate drug discovery programs.^23^

 During the last 5 years, this technique has achieved a significant development and maturity degree, being used extensively for the search of new inhibitors^24^ and for the identification of new therapeutic targets,^25^ highlighting, once again, its importance as a tool for drug discovery processes. Finally, comparative analyses of co-occurrence networks showed that ABPP is currently being extensively applied for understanding drug resistance mechanisms,^26^ developing tools for polyubiquitination,^27^ and searching for new molecular therapeutic targets.^28^ Although this type of analysis is not extensively utilized in the literature, it represents one of the significant advantages of bibliometric analysis. By comparing all articles with the most recently reported ones, this approach provides valuable insights into the current trends and direction of the field, which may eventually lead to the prediction of future research directions.

## Future directions and Conclusions: An outlook derived from this bibliometric analysis

 The sequencing of the human genome has yielded a tremendous amount of information about the genetic makeup of cells.^29^ However, despite the existence of around 20 000 genes in the human genome, the functions of only a fraction of the resulting proteins are fully understood.^30^ This highlights the challenge of translating genomic data into a comprehensive understanding of cellular functions.

 ABPP is a cutting-edge chemical proteomics approach that offers quantitative and unbiased detection of enzymatic activity by profiling cellular and tissue activity, while it also offers a complementary solution to traditional gene expression analysis and serves as an effective tool in decoding the abundance of genomic information.^31^ This allows researchers to gain a deeper understanding of the functional state of an enzyme, beyond its mere abundance. The design and use of ABPs and AfBPs is crucial in ABPP.^21^ Numerous probes are currently available for studying enzymes, including kinases, proteases, glycosidases, phosphatases, and oxidoreductases. ABPP has been successful in identifying enzymatic activities related to various diseases^32^ and facilitating functional studies in biological processes, such as neurotransmission,^33^ neurodegenerative diseases,^34^ and tumor and immune signaling.^35^

 However, ABPP faces significant obstacles, such as poor specificity and sensitivity, with many human enzymes lacking selective chemical ligands, and some considered undruggable.^36^ Strategies such isoTOP-ABPP (Isotope Tagging of Optimized Probes for Activity-Based Protein Profiling),^37^ have been developed to overcome these limitations. This technology uses stable isotopes to enhance the sensitivity and specificity of ABPP and to facilitate the analysis and quantification of protein interactions in complex biological samples.

 ABPP is a multidisciplinary field that demands collaborative interdisciplinary efforts. It is essential to acknowledge that no single design strategy is adequate for all research needs, and the pursuit of more and better ABPs and AfBPs remains a challenging task.^21^ Another area that holds great promise for the future is the creation of fluorescent ABPs, which can be used in both human and animal health contexts. Consequently, one of the crucial challenges in contemporary biomedical research is to develop non-invasive, spatially controlled ABPs for real-time imaging of specific protein biomarkers in whole organisms.^11^

 On the other hand, the integration of multi-omics (including metabolomics, peptidomics, and proteomics) and ABPP is essential for gaining a comprehensive understanding of biological systems.^38^ By combining these different omics approaches, a more complete picture of the biological processes can be obtained. ABPP, in particular, provides valuable insights into protein functionality, adding to the knowledge gained from genomics and proteomics. Thus, the integration of these approaches is crucial for advancing our understanding of biological systems.

 In conclusion, this analysis highlights the significant growth and versatility of ABPP platforms, cementing their position as a valuable tool in drug discovery. With the ongoing advancements in quantitative proteomics and multi-omics platforms, and the continual development of novel ABPs and AfBPs, it is anticipated that ABPP will continue to play a pivotal role in advancing the drug discovery process in the future, enabling the identification of potential therapeutic targets, and aiding in the optimization of drug candidates.

## Acknowledgments

 EP was supported, in part, by a grant from the COFUND (European Union and Durham University) - Durham International Fellowships for Research and Enterprise, Junior Research Fellowships (JRF) scheme, and by a grant from the UKRI - Global Challenges Research Fund. “A Global Network for Neglected Tropical Diseases”.

## Competing Interests

 The author declares that he has no known competing financial interests or personal relationships, which have, or could be perceived to have, influenced the work reported in this article.

## Ethical Approval

 Not applicable.
